# Aloe Vera Functionalized Magnetic Nanoparticles Entrapped Ca Alginate Beads as Novel Adsorbents for Cu(II) Removal from Aqueous Solutions

**DOI:** 10.3390/nano12172947

**Published:** 2022-08-26

**Authors:** Surbhi Lilhare, Sunitha B. Mathew, Ajaya Kumar Singh, Sónia A. C. Carabineiro

**Affiliations:** 1 Department of Chemistry, Govt. V. Y. T. PG Autonomous College, Durg, Chhattishgarh 491001, India; 2 School of Chemistry & Physics, Westville Campus, University of KwaZulu-Natal, Durban 4000, South Africa; 3 LAQV-REQUIMTE, Department of Chemistry, NOVA School of Science and Technology, Universidade NOVA de Lisboa, 2829-516 Caparica, Portugal

**Keywords:** copper ion adsorption, magnetic nanoparticles, aloe-vera functionalized alginate beads, adsorption isotherm, spectrophotometric method

## Abstract

CABs (Ca alginate beads), AVCABs (Aloe vera Ca alginate beads), and AVMNCABs (Aloe-vera functionalized magnetic nanoparticles entrapped Ca alginate beads) were developed as adsorbents for the removal of Cu(II) from aqueous solutions. The materials were characterized using Fourier-transform infrared (FTIR) spectroscopy, high-resolution scanning electron microscopic (HR-SEM) analysis, X-ray diffraction (XRD), energy-dispersive X-ray (EDX) spectroscopy, and a vibrating-sample magnetometer (VSM). The effect of several parameters, such as pH, time, temperature, adsorbent dose, etc., were investigated. The adsorption isotherm of Cu(II) was adjusted best to the Langmuir model. The maximum adsorption capacities were 111.11 mg/g, 41.66 mg/g, and 15.38 mg/g for AVMNCABs, AVCABs, and CABs, respectively. The study of the adsorption kinetics for Cu(II) ions on beads followed a pseudo-second-order kinetic model, with a very good correlation in all cases. The adsorption studies used a spectrophotometric method, dealing with the reaction of Cu(II) with KSCN and variamine blue.

## 1. Introduction

Cu(II) is essential for the human body, but excessive intake can damage organs and, in extreme cases, lead to death. Copper is commonly used in roofing, plumbing, electroplating, utensils, pesticides, fertilizers, and medicines. It may also result from mining and industrial wastes. Exposure to high levels of copper or its intake through contaminated food, air, and water is dangerous [1,2]. The ingestion of high doses of copper can cause severe mucosal corrosion and irritation, central nervous system damage, followed by depression and intense capillary damage. It can also cause gastrointestinal irritation and the necrosis of liver and kidney tissues, leading to hepatic and renal damage due to chronic poisoning. The most serious situations are diseases such as leukemia, hepatitis, and Wilson’s disease, among others. The World Health Organization recommends 1 mg L^−1^ as the maximum acceptable limit of Cu(II) in drinking water [3].

Several methods, such as adsorption, reverse osmosis, chemical precipitation, ion exchange, membrane filtration, evaporation, and electrochemical procedures [4,5,6,7], have been reported for the treatment of wastewater. Among those, adsorption is promising for removing Cu(II) ions, given its operational ease, high efficiency, low cost, and good recyclability. Several types of adsorbents, such as zeolites [8], biomasses [9,10,11,12,13,14,15], fly ash [16], activated carbon [17,18,19], chitosan [20], polymeric hybrid sorbents [21,22,23,24,25,26], and agriculture wastes [27] have been reported. A large number of the latter, namely orange peels [28], palm shells [29], sawdust [30], hazelnut shells [31], rice husk [32], walnut shells [33], etc., have been used as cheap adsorbents for removing Cu(II) from aqueous solutions. 

Magnetic nanoparticles can be entrapped inside various organic and inorganic stabilizers, such as activated carbon [17,18,19], chitosan [34,35,36,37], alginate biopolymer [38,39], and β-cyclodextrin [40,41] to minimize their aggregation, enhance their adsorption efficiency, and enable the easy separation from the matrix. Sodium alginate is a water-soluble and commonly used natural polysaccharide with wide uses due to its non-toxic, inexpensive, biocompatible, biodegradable, emulsion stabilizing, and bioadhesive nature [42,43]. The alginates have the ability to form gels in the presence of calcium ions (Ca^2+^), which occurs through the interaction and cross-linking between the L-guluronic acid (G) of alginate and the calcium ion (Ca^2+^) in solution. 

Aloe vera is a tropical, medicinal plant with a polysaccharide-rich matrix. It contains amino acids, lignin, salicylic acid, enzymes, vitamins, minerals, several carbohydrates, etc. Such molecules have functional groups that are able to bind with metals [44,45].

In this study, bio-sorbent AVMNCABs (Aloe-vera functionalized magnetic nanoparticles on Ca alginate beads) were prepared by mixing the pulp of Aloe vera leaves, a natural ingredient, with alginate and magnetic nanoparticles. Its efficiency for Cu(II) removal from aqueous solutions was compared with CABs (calcium alginate beads) and AVCABs (Aloe vera calcium alginate beads) using a simple spectrophotometric method. The performance and reusability of the adsorbents were investigated, and the influence of the temperature, time, and pH was studied in batch conditions. The thermodynamic and kinetic results revealed that the materials had advantages, such as a high adsorption capacity, simplicity, inexpensiveness, and good reproducibility. Adsorption isotherm models were used to explore the possibility of applying the synthesized adsorbents to the removal of Cu(II) ions from aqueous solutions. The study was carried out by a simple spectrophotometric procedure established in our laboratory and using potassium thiocyanate and variamine blue [46].

## 2. Materials and Methods

### 2.1. Reagents

The reagents used were of analytical grade. Double distilled water was used in all of the experiments. Copper sulfate (CuSO_4_), ferrous chloride dihydrate (FeCl_2_∙2H_2_O), ferric chloride hexahydrate (FeCl_3_∙6H_2_O), and potassium thiocyanate (KSCN) were obtained from Merck (Mumbai, India). Variamine blue (VB) [0.05%], sodium alginate (C_6_H_9_NaO_7_)—used for obtaining the alginate beads, and calcium chloride (CaCl_2_), which was used for cross-linking the alginate beads—were obtained from Sigma-Aldrich (Mumbai, India). Moreover, 0.1 M sodium hydroxide (NaOH) and 0.1 M hydrochloric acid (HCl) were also prepared.

The Aloe vera leaves were collected from a nearby area, washed several times with double distilled water to remove dust and other impurities, and then cut into small pieces, followed by extraction of the gel. 

### 2.2. Equipment

X-ray diffraction (XRD) analysis was carried out in an Expert-Pro PW3064/60 apparatus from 5° to 80° (Almelo, Netherlands). A Thermo Nicolet Avtar 370 Fourier-transform infrared (FT-IR) spectrometer (Tokyo, Japan) was used to record the infrared spectra in the 500–4000 cm^−1^ range with the help of KBr pellets. Scanning Electron Microscopic (SEM) images were obtained in a Jeol 6390LA/OXFORD XMX N (Tokyo, Japan) to analyze the surface morphology of the adsorbents. Energy-Dispersive X-ray (EDS/EDX) spectroscopy analysis (Oxford instruments, Abingdon, UK) was utilized to obtain the elemental composition. A vibrating-sample magnetometer (VSM) (Lake Shore 7410 model Westerville, OH, USA) was used to obtain the magnetization curve of the adsorbents. A Systronics ultraviolet-visible (UV-Vis) spectrophotometer-117 (Carry 50 scan, Varian, East Lyme, CT, USA) was used to measure the absorbance. The pH measurements were carried out on a digital Systronics pH meter (112 model, Ahmedabad, India). The temperature was kept constant using a thermostatic water bath.

### 2.3. Preparation of Beads

#### 2.3.1. Synthesis of Ca Alginate Beads (CABs)

Sodium alginate (SA) (1.5 g powder) was added to 50 mL of double distilled water. A viscous solution was obtained, which was added dropwise into a CaCl_2_ (2%, *w*/*v*) solution with gentle stirring, resulting in the formation of the spherical bio-sorbent. The beads were then left for 24 h in the CaCl_2_ solution to become stable and rinsed three times with distilled water to eliminate excess Ca(II) and stored in distilled water for subsequent use.

#### 2.3.2. Synthesis of Aloe-Vera Calcium Alginate Beads (AVCABs)

The Aloe vera (AV) gel (0.25 g) was added to the viscous solution containing sodium alginate (1.5 g in 50 mL of distilled water). The solution thus obtained was added dropwise into CaCl_2_ (2%, *w*/*v*) aqueous solution with stirring. The beads were then left for 24 h in the CaCl_2_ solution to become stable. The gel beads were then washed three times with distilled water and stored in clean distilled water for further use.

#### 2.3.3. Synthesis of Aloe-Vera Functionalized Magnetic Nanoparticles on Ca Alginate Beads (AVMNCABs)

The Fe_3_O_4_ nanoparticles (IN) were obtained by co-precipitation, mixing ferric chloride (Fe^2+^) and ferrous chloride (Fe^3+^) in a molar ratio of 2:1, followed by the addition of 1.5 M NH_4_OH solution at room temperature (~30 °C) under vigorous stirring. The nanoparticles were obtained as a black precipitate that was magnetically separated and then washed three times with double distilled water and dried at 200 °C for 2 h. 

**Figure 1:**
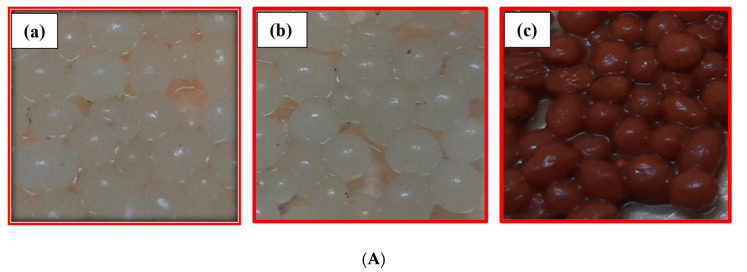

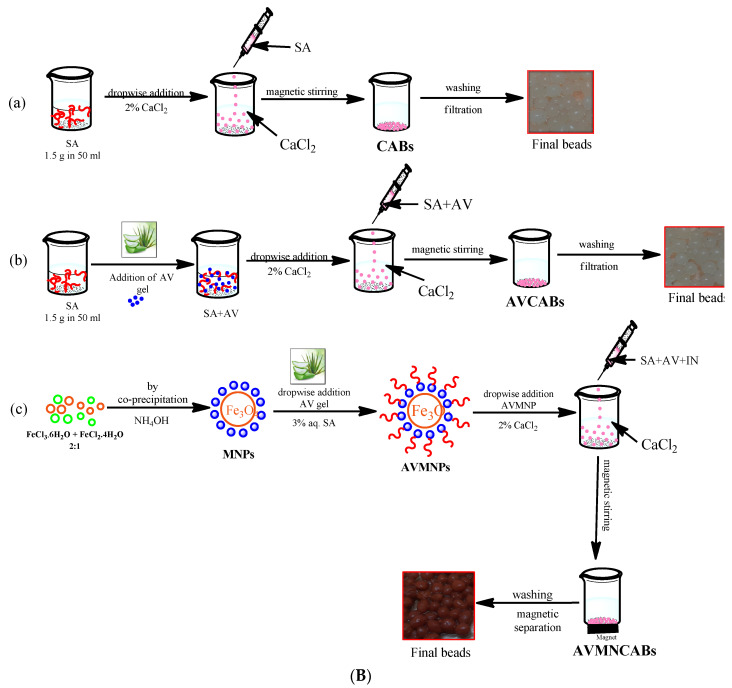
(**A**) Images of the (**a**) CABs, (**b**) AVCABs and (**c**) AVMNCABs gel beads (approximate size of beads: 2–3 mm); (**B**) Synthesis of (**a**) CABs, (**b**) AVCABs, and (**c**) AVMNCABs gel beads.

The Fe_3_O_4_ nanoparticles (0.05 g) were added to the viscous solution containing 0.25 g of Aloe vera gel in a 3% aqueous sodium alginate solution. Later, the above mixture was added dropwise to the CaCl_2_ solution, leading to the formation of AVMNCABs. The beads were then left for 24 h in the CaCl_2_ solution to become stable. Then, they were washed three times with double distilled water and stored for later use in distilled water. The beads became red–brown due to the modified magnetic nanoparticles. The representative images of the gel beads (CABs, AVCABs, and AVMNCABs) are displayed in Figure 1A (their approximate size was 2–3 mm). The synthesis of the gel beads is represented in Figure 1B.

### 2.4. Spectrophotometric Determination of Cu (II)

After the adsorption, the beads were separated from the liquid by filtration, and the concentration of Cu(II) was spectrophotometrically determined using potassium thiocyanate and variamine blue. Then, 5 mL of filtrate, 0.5 mL of potassium thiocyanate (0.2 N), and 0.6 mL of a 0.05% variamine blue solution were added, which resulted in the oxidation of the leucoform of variamine blue to variamine blue (violet color), with a maximum absorbance at 550 nm. The concentration of the Cu(II) was obtained from the calibration curve [46].

### 2.5. Batch Adsorption Experiments

The adsorption performance of the gel beads for removing copper from aqueous solutions was investigated using a batch adsorption approach. The effects of pH (2–7), adsorbent amount (0.05–0.6 g), time of contact (5–180 min), and initial ion concentration (10–90 mg L^−1^) on the removal efficiency were studied. The pH was adjusted by using 0.1 M HCl/0.1 M NaOH. A fixed volume of Cu(II) ion solution (10 μg mL^−1^) was shaken with 0.2 g of CABs, AVCABs, and AVMNCABs at a pH of 4 and at 35 °C for 120 min. The adsorption isotherms were obtained by using aqueous solutions of Cu(II) with several concentrations. After the adsorption, the adsorbents were separated by filtration, and the Cu (II) amount in the filtrate was determined by UV-vis at 550 nm using potassium thiocyanate and variamine blue. The materials were characterized using SEM-EDX, FTIR, XRD, and VSM. The percentage of removal, adsorption capacity at equilibrium (*q_e_*), and at a given time *t* (*q_t_*) were calculated using Equations (1)–(3), respectively:

(1) $$R=\frac{C_{0}\;-\;C_{e}}{C_{0}}$$

(2) $$q_{e}\;\left(\%\right)=\frac{(C_{0}\;-\;C_{e)\;V}}{m}$$

(3) $$q_{t}\;\left(\%\right)=\frac{(C_{0}\;-\;C_{t)\;V}}{m}$$

where *C*_0_, *C_e_*, and *C_t_* are the initial, equilibrium, and at a given *t* time concentrations of Cu(II) ions (μg mL^−1^), respectively, *m* is the adsorbent mass (g), and *V* is the solution volume (L). 

### 2.6. Desorption

**Figure 2:**
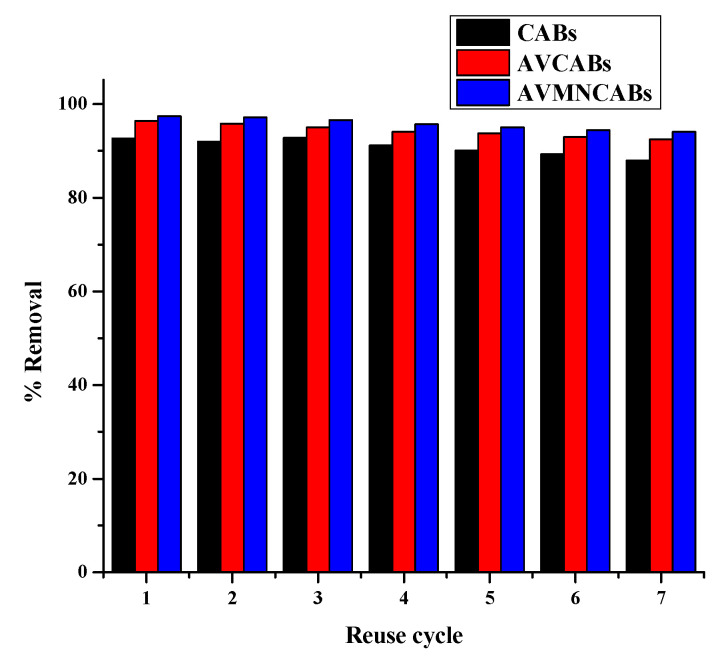
Reusability of CABs, AVCABs, and AVMNCABs up to 7 cycles.

The desorption was carried out using 2 mL of a 0.05 M HNO_3_ solution as the desorption agent. The adsorbent was washed with double distilled water three times, and its reusability was assessed. The adsorbents exhibited good adsorption–desorption performance and could be reused up to 7 cycles (Figure 2). The desorption (%) was calculated by:

(4) $$\mathrm{Desorption}\left(\%\right)=\frac{\mathrm{Desorbed}\;\mathrm{quantity}\;\mathrm{of}\;\mathrm{metal}}{\mathrm{Adsorbed}\;\mathrm{quantity}\;\mathrm{of}\;\mathrm{metal}}\times 100$$

92.7%, 96.4%, and 97.4% desorption were obtained for CABs, AVCABs, and AVMNCABs, respectively.

## 3. Results and Discussion

### 3.1. Adsorbent Characterization

#### 3.1.1. X-ray Diffraction (XRD)

**Figure 3:**
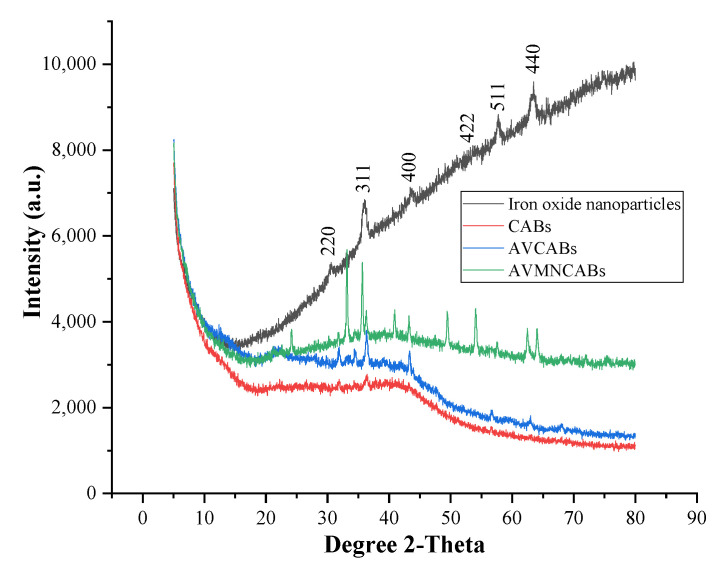
XRD patterns of iron oxide (Fe_3_O_4_) nanoparticles, CABs, AVCABs, and AVMNCABs, with the characteristic peaks of Fe_3_O_4_ identified.

The XRD diffractograms of the iron oxide nanoparticles, CABs, AVCABs, and AVMNCABs show several characteristic peaks (Figure 3). The XRD pattern of CABs shows broad peaks at 2θ values of 31.80° and 36.61°, while AVCABs show comparatively sharp peaks at 2θ values of 31.87°, 34.33°, 36.25°, 43.37°, 56.69°, and 62.97°. The XRD pattern of the iron oxide nanoparticles shows broad peaks at 2θ = 30.20°, 36.24°, 43.21°, 53.74°, 57.23°, and 62.90°, identical to the standard JCPDS data [47] for Fe_3_O_4_, which can be indexed to (220), (311), (400), (422), (511), and (440) planes. The Aloe vera-alginate beads containing iron oxide nanoparticles (AVMNCABs) show six characteristic peaks at 2θ values of 30.2°, 35.67°, 43.21°, 54.08°, 57.62°, and 62.51°, identical to that of the planes of Fe_3_O_4_ [48]. The narrow, sharp peaks of AVMNCABs indicate the small crystallites and ultra-fine nature of the particles and indicate the spinal structure.

The average crystal size of the CABs, AVCABs, and AVMNCABs were determined using Debye–Scherrer’s Equation [49]:

(5) $$D=\frac{K\cdot \lambda }{\beta \cdot cos\theta }$$

where D is the average size of crystallites in Å, *θ* is the diffraction angle, *β* is the FWHM (full width at half maximum) of the peak, *λ* is the X-ray wavelength (1.54 Å), and *K* is a constant (*K* = 0.9 Å). Table 1 shows the particle sizes of CABs, AVCABs, and AVMNCABs: 0.072 nm, 0.131 nm, and 0.022 nm, respectively.

#### 3.1.2. Fourier Transform Infrared Spectroscopy (FTIR)

**Figure 4:**
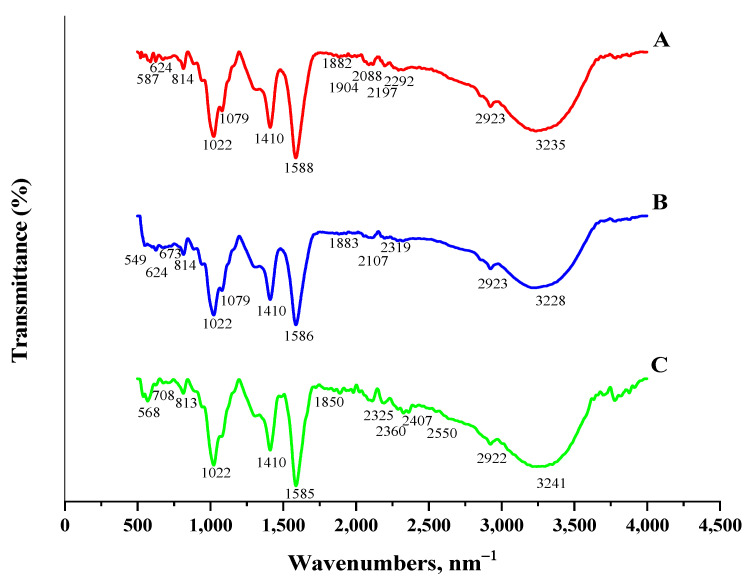
FTIR spectra of (**A**) CABs, (**B**) AVCABs, and (**C**) AVMNCABs before adsorption.

The FTIR spectra of the CABs, AVCABs, and AVMNCABs are displayed in Figure 4. Calcium alginate shows characteristic peaks at 3235 and 2923 cm^−1^, attributed to O-H stretching and C-H stretching vibrations. The bands at 1588 and 1410 cm^−1^ are due to the asymmetric and symmetric vibrations of C=O of the COO^−^ group. The peak around 1079 cm^−1^ corresponds to C-O, C-C, and C-O-C stretching vibrations, and the sharp peak at 1022 cm^−1^ is due to C-C and C-O-C stretching [50]. The spectrum of the AVCABs shows shifts in the position and intensity of the bands due to -OH stretching (3228 cm^−1^) and asymmetric C=O stretching vibrations (1586 cm^−1^). In the case of AVMNCABs, the peaks shift to 3241, 2922, and 1585 cm^−1^. The intensity of the peaks decreases due to the chelation of OH and COO^−^ to the Fe^3+^ ion. An additional peak at 568 cm^−1^ is observed, which can be ascribed to Fe-O vibration. 

#### 3.1.3. Vibrating Sample Magnetometer (VSM)

**Figure 5:**
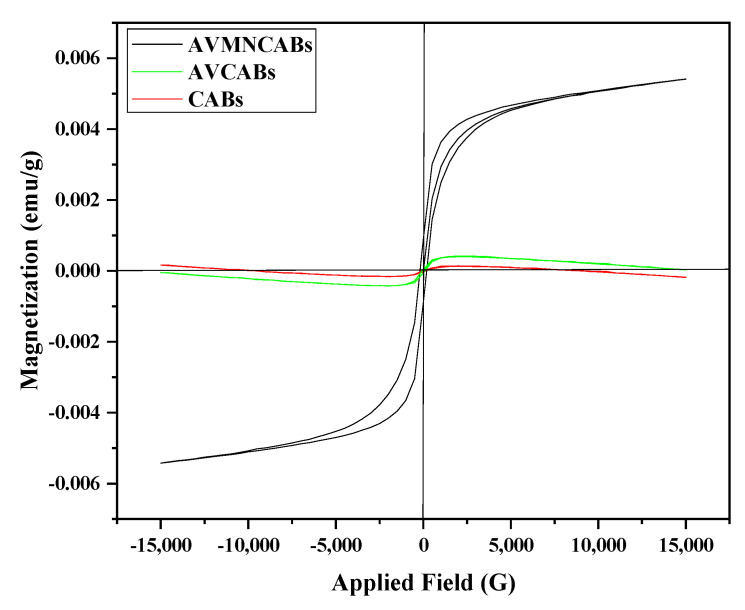
Vibrating sample magnetization (VSM) of CABs, AVCABs, and AVMNCABs (at room temperature).

This technique was used to assess the magnetic properties of the materials, such as retentivity (M_R_), coercivity (H_C_), and saturation magnetization (M_S_). The hysteresis loop describing the magnetization of CABs, AVCABs, and AVMNCABs is shown in Figure 5. The saturation magnetization measures the material magnetization extent in an external magnetic field. The coercivity is the intensity of the magnetic field needed to reduce the material magnetization to zero after the sample has been magnetized. The values of M_S_ are 6.77 × 10^−3^, 4.89 × 10^−3^, and 1.08 × 10^−1^ emu/g for CABs, AVCABs, and AVMNCABs, respectively, indicating the enhancement of the magnetic properties of AVMNCABs after the incorporation of iron nanoparticles, compared to CABs and AVCABs. The value of H_S_ is 193.15 O_e_ for AVMNCABs, showing the ferromagnetic nature of the AVMNCABs, whereas the other adsorbents are non-magnetic. A decrease in M_S_ has been reported in several adsorbents [51,52,53]. The obtained low value of M_S_, compared to iron nanoparticles, can be explained by the presence of a non-magnetic substance in the adsorbent matrix. The appreciable magnetic properties of AVMNCABs, compared to CABs and AVCABs, enable the easy removal by the first adsorbent in less time.

#### 3.1.4. Scanning Electron Microscopy (SEM)

**Figure 6:**
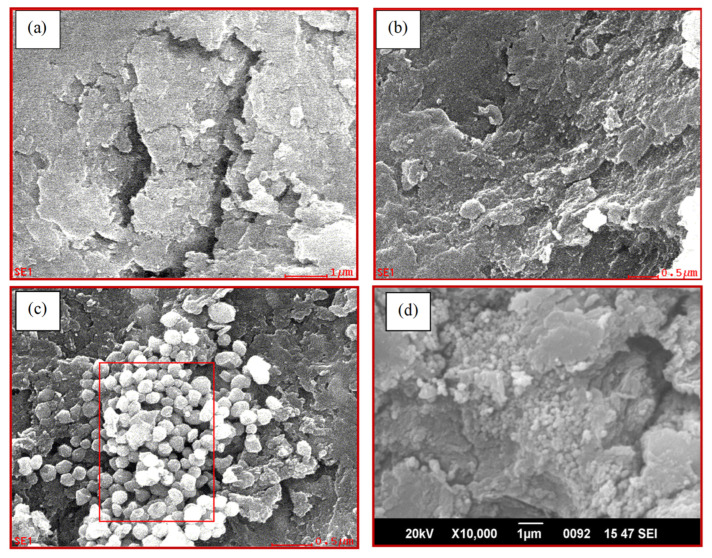
SEM images of (**a**) CABs, (**b**) AVCABs, and (**c**) AVMNCABs before adsorption and (**d**) AVMNCABs after adsorption.

The morphological features of the synthesized adsorbents were investigated by SEM. The images of CABs (a), AVCABs (b), and AVMNCABs (c) show the surface morphology of the various alginate beads (Figure 6). AVMNCABs are shown to have a more irregular, uneven structure, with a large surface area available for adsorption compared to the other materials. Figure 6 shows that the surface of the AVMNCABs has more pores than CABs and AVCABs, indicating the enhanced adsorption capacity of the former material (Figure 6a–c). Figure 6d shows that the surface of AVMNCABs has less porosity after adsorption, as most of the available pores found in beads are covered, causing the surface of the beads to be more saturated and smother.

#### 3.1.5. Energy Dispersive X-ray (EDX) Spectroscopy

**Figure 7:**
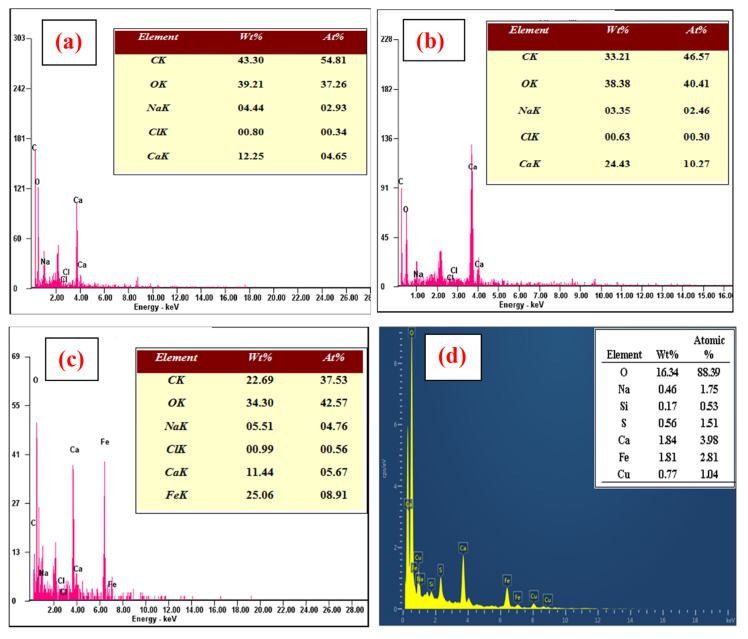
EDX spectra of (**a**) CABs, (**b**) AVCABs, and (**c**) AVMNCABs before adsorption; and (**d**) AVMNCABs after adsorption.

EDX was used to determine the composition of the samples. As shown in Figure 7a, CABs contain C (43.3%), O (39.21%), Na (4.44%), and Ca (12.25%). Figure 7b shows that the AVCABs have C (33.21%), O (38.38%), Na (3.35%), and Ca (24.43%), and Figure 7c indicates that AVMNCABs (after adsorption) comprise C (22.69%), O (34.30%), Na (5.51%), Ca (11.44%), and Fe (25.06%), which confirms the incorporation of iron nanoparticles onto the alginate beads. Figure 7d clearly shows the presence of Cu in the nanocomposite matrix of AVMNCABs, demonstrating the efficiency of the beads in removing Cu(II) from the water.

### 3.2. Effect of pH

**Figure 8:**
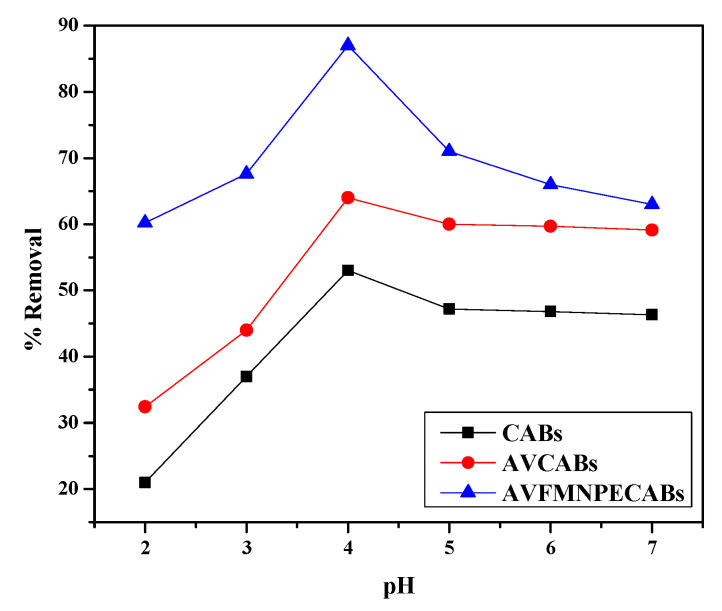
pH effect on Cu(II) removal for an initial concentration of 10 µg mL^−1^; 0.2 g adsorbent; 1 h.

The pH of the solution is a crucial factor in the removal of Cu(II). The pH was adjusted using different quantities of 0.1 M HCl or 0.1 M NaOH solutions. The influence of pH on the removal of Cu(II) was tested by changing the pH from 2 to 7 using an initial Cu(II) concentration of 10 mg L^−1^, 0.2 g adsorbent, and a 1 h contact time. Figure 8 shows that the removal (%) increases with pH increase until pH 4. Above that value, a reduction in removal is found in all the cases. 

**Figure 9:**
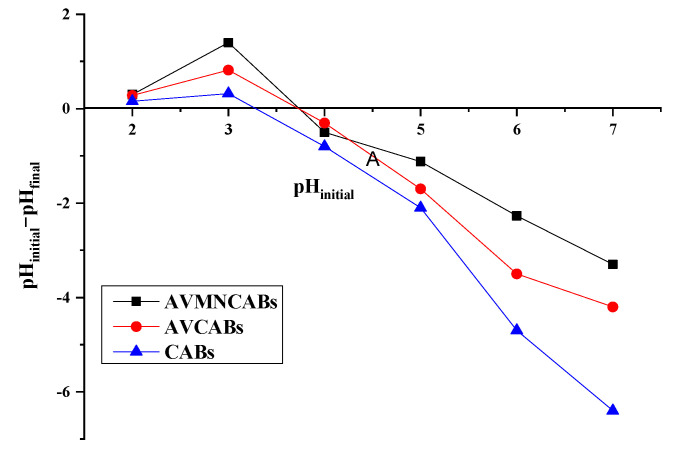
Point of zero charge (pH_pzc_) of AVMNCABs, AVCABs, and CABs.

The point of zero charge (pH_pzc_) was 3.72 for AVMNCABs and AVCABs and 3.32 for CABs (Figure 9). For pH values below pH_pzc_, the surface of the adsorbent has positive charges. However, for pH > pH_pzc_, the surface becomes negative, which facilitates the electrostatic attraction of the copper(II) ion. The maximal adsorption of Cu(II) was observed at pH 4, which is above the pH_pzc_ value [54]. 

At lower pH values, the adsorbents are protonated; that is, they have a positive surface charge, which leads to strong repulsion with the also positively charged Cu(II) ions. Furthermore, for pH > 4, the surface active sites of the adsorbents are unprotonated, and hence, the surface has a negative charge, which increases the adsorption of Cu(II). At highly-basic pH, Cu(II) ions can precipitate in the form of insoluble hydroxides, lowering the adsorption. Moreover, the competition between Cu(II) and H^+^ can also decrease adsorption. The maximum removal efficiencies for CABs, AVCABs, and AVMNCABs were 53%, 64%, and 87%, respectively, at pH 4. 

### 3.3. Effect of Adsorbent Amount

**Figure 10:**
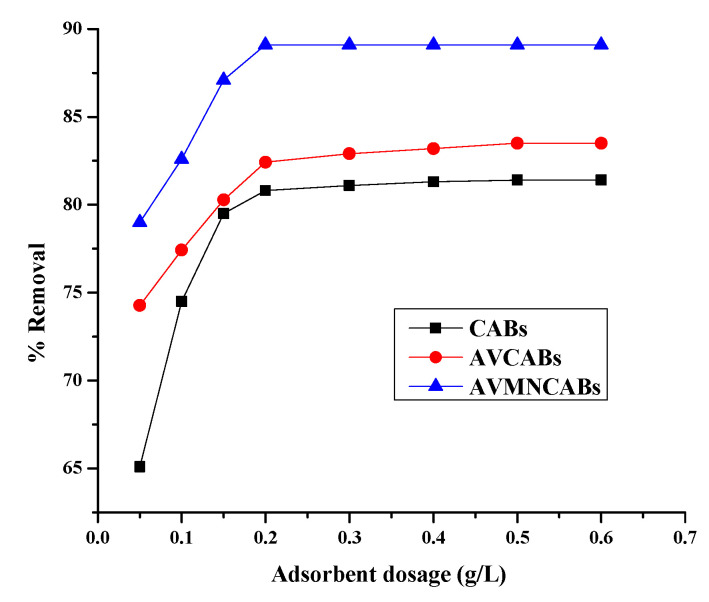
Variation of the removal of Cu(II) with adsorbent dose (10 µg mL^−1^ Cu(II), 1 h, 30 °C, pH 4).

The adsorption capacity of the materials was revealed by the variation of the adsorbent amount from 0.05 to 0.6 g (Figure 10). After a certain dose, the removal shows no significant changes. The amount of Cu(II) adsorbed by CABs, AVCABs, and AVMNCABs increased from 65.1 to 81.4%, 74.28 to 83.5%, and 79 to 89.1%, respectively, for adsorbent amounts ranging from 0.1 to 0.4 g (CABs), 0.1 to 0.5 g (AVCABs), and 0.1 to 0.2 g (AVMNCABs). The increase in the removal efficiency with the increase in the amount of adsorbent can be correlated with a higher number of active sites on the adsorbent surface, which are accessible to the metal ions. 

### 3.4. Effect of Time

**Figure 11:**
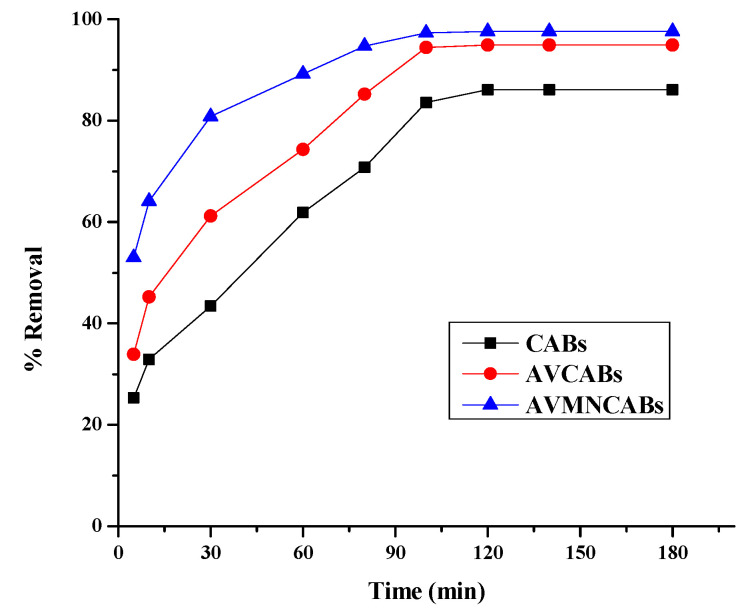
Effect of time on the removal of Cu(II) (10 µg mL^−1^ Cu(II), 0.2 g adsorbent; 30 °C, pH 4).

The influence of time on the removal of Cu(II) was determined from 5–180 min at pH 4, and the results can be found in Figure 11. The removal ranged from 25.3 to 97.6% in the time span of 5–120 min. The minimum and maximum efficiencies for CABs, AVCABs, and AVMNCABs, were 25.3–86.1%, 33.9–94.6%, and 53–97.6%, respectively, for 5 to 120 min. Initially, the adsorption process was quite fast but slowed down with time. The initial higher rate of removal could be caused by the relatively high Cu(II) concentration and a large number of vacant active surface sites on the adsorbent. The lowering of the removal rate with time can be ascribed to a decrease in the availability of the adsorbent active sites. 

### 3.5. Effect of Copper Ion Concentration

**Figure 12:**
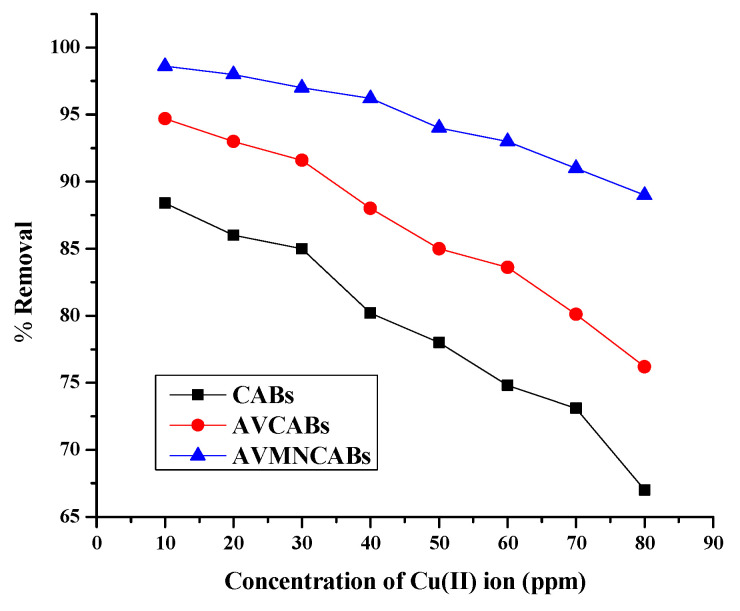
Effect of the initial Cu(II) concentration on the adsorption (0.2 g adsorbent, 120 min, 30 °C, pH 4).

Cu(II) adsorption was investigated for the previously found optimum time (120 min), pH 4, 10 to 80 µg mL^−1^ copper ion concentration. An increase in the initial value leads to an adsorption decrease. Figure 12 shows that the maximal removal of Cu(II) was 88.4% (CABs), 94.7% (AVCABs), and 98.6% (AVMNCABs). More adsorption sites are available for lower Cu(II) concentrations. As the number of Cu(II) ions increases, for higher concentrations, less active sites are available for adsorption. Hence, adsorption depends on the initial concentration of Cu(II). As this concentration increases, more active sites are covered, and Cu(II) ions have greater competition for the surface of the adsorbent [55].

### 3.6. Adsorption Isotherms

Various models of adsorption isotherms were tested to assess the adsorption capacity under optimized conditions. The acquired data were applied to the Freundlich [56], Langmuir [57], and Temkin [58] models. The adsorption parameters obtained for CABs, AVCABs, and AVMNCABs for removing Cu(II) are in Table 2.

**Figure 13:**
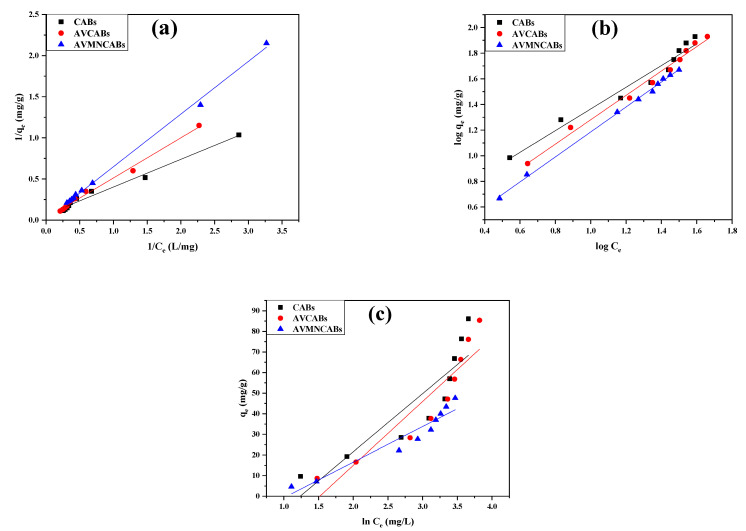
Adsorption isotherms: (**a**) Langmuir, (**b**) Frendlich, and (**c**) Temkin for CABs, AVCABs, and AVMNCABs.

The Langmuir isotherm model is characterized by the equation:

(6) $$\frac{1}{q_{e}}=\frac{1}{q_{m}}+\frac{1}{K_{m}q_{m}}\;\cdot \;\frac{1}{C_{e}}$$

where: *K_m_* is the Langmuir adsorption constant (L/mg), *q_m_* is the adsorbent maximum adsorption capacity (mg/g); *C_e_* and *q_e_* are the equilibrium concentration of Cu(II) ion and equilibrium adsorption capacity (mg/L), respectively. The 1*/q_e_* versus 1*/C_e_* plot is depicted in Figure 13A.

The separation factor, constant, and equilibrium parameter *R_L_* were calculated with

(7) $$R_{L}=\frac{1}{1+K_{L}C_{0}}$$

*R_L_* infers if the adsorption is irreversible (for *R_L_* = 0), favorable (for 0 < *R_L_* < 1), linear (for *R_L_* = 1), or not favorable (for *R_L_* > 1). In the present case, the value of *R_L_* is much smaller than 1, confirming that Cu(II) adsorption is a favorable process [59,60,61].

The Freundlich model depicting multilayer adsorption is expressed as:

(8) $$\log\;q_{e}=\log K_{F}+\frac{1}{n}\log C_{e}$$

where: *n* and *K_F_* (L/mg) are the Freundlich constants, indicative of the intensity and adsorption capacity, respectively; *q_e_* and *C_e_* are the equilibrium adsorption capacity (mg/g) and equilibrium concentration of Cu(II), respectively. *K_F_* and 1/*n* values can be obtained from the intercept and slope of the log *q_e_* versus log *C_e_* plot represented in Figure 13B.

The Temkin isotherm model is centered on the surface coverage. The adsorption energy decreases linearly with coverage. This isotherm is expressed by Equation (9):

(9) $$q_{e}=B_{1}lnk_{T}+B_{1}lnc_{e}$$

where: *B*_1_ = *RT/b*; *b* is the Temkin constant (J/mol); *T* is the temperature (K); *R* is the gas constant (8.314 J/mol K); *B*_1_ is the constant related with the adsorption heat (J/mol); *K_T_* is the constant of equilibrium binding (L/g). The determination of *K_T_* and *B*_1_ can be performed by the *q_e_* versus *ln C_e_* plot, as shown in Figure 13C.

### 3.7. Adsorption Kinetics

**Figure 14:**
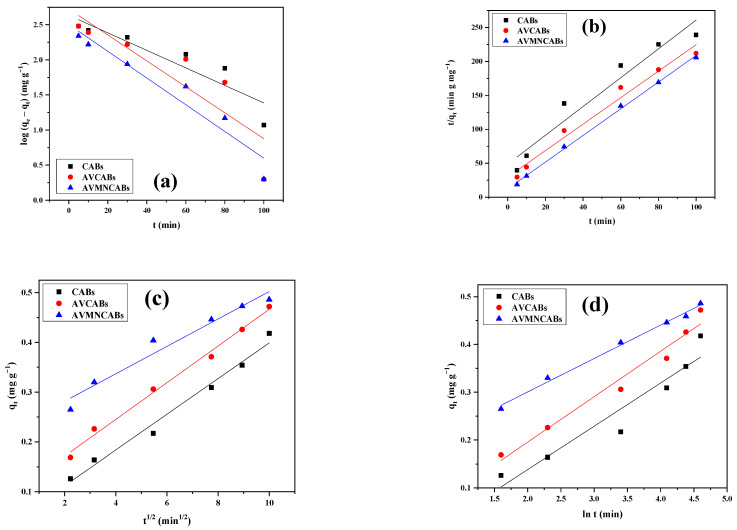
(**a**) Kinetic fittings for pseudo-first order, (**b**) pseudo-second order, (**c**) intra-particle diffusion, and (**d**) Elovich models.

The pseudo-first-order [62], pseudo-second-order [63], Elovich kinetics [64], and intra-particle diffusion [65] models were used to investigate the adsorption kinetics. The effect of the contact time on the adsorption is presented in Figure 14 and Table 3. 

The pseudo-first-order, pseudo-second-order, Elovich kinetics, and intra-particle diffusion follow Equations (10)–(13).

(10) $$\log\;(q_{e}-q_{t})=\log\;q_{e}-\frac{k_{1}t}{2.303}$$

where: *q*_e_ and *q_t_* are the quantity of copper adsorbed (mg g^−1^) at equilibrium and at a given time *t* (min), respectively; *k*_1_ (min^−1^) is the pseudo-first-order rate constant. The log (*q_e_* − *q_t_*) versus *t* plot is shown in Figure 14A.

(11) $$\frac{1}{q_{t}}=\frac{t}{k_{2}q_{e}{}^{2}}-\frac{1}{q_{e}}$$

where: *k*_2_ (g/mg min^−1^) is the pseudo-second-order rate constant; *q*_e_ and *q_t_* are the same as above. *t/q_t_* versus *t* plot is shown in Figure 14B. The highest correlation coefficient was obtained for pseudo-second-order kinetics.

The intra-particle diffusion model centered on the diffusion mechanism, suggested by Weber and Morris, is given by:

(12) $$q_{t}=K_{d}\;t^{1/2}+C$$

where: *K_d_* is the pores diffusion rate constant (mg/g min^1/2^); *C* is the intercept (mg/g). The *d_t_* versus *t*^1/2^ plot is shown in Figure 14C.

The Elovich equation is generally used in chemisorption and satisfactorily applies to the chemisorption process; it is expressed as:

(13) $$q_{t}=\alpha +\beta \ln t$$

where: *q_t_* (mg/g) is the amount of copper(II) adsorbed at time *t* (min); *α* (mg g^−1^ min^−1^) and *β* (g mg^−1^) are constants, respectively, obtained from the intercept and the slope of *q_t_* versus ln *t* linear plot, as shown in Figure 14D.

### 3.8. Thermodynamic Parameters

**Figure 15:**
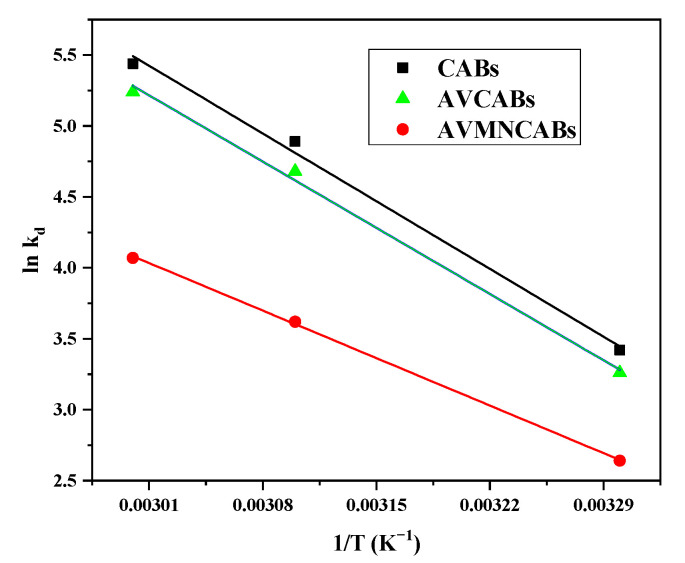
Van’t Hoff’s plot for Cu(II) adsorption.

The thermodynamic parameters, namely the free energy of Gibbs (Δ*G*, kJ mol^−1^), changes in enthalpy (Δ*H*, kJ mol^−1^), and entropy (Δ*S*, J mol^−1^ K^−1^) for the adsorption of Cu(II) on various beads was determined using the equilibrium data obtained at different temperatures using the Gibbs and Van’t Hoff equations [66]:

(14) $$\Delta G=-RT\ln K_{d}$$

(15) $$\ln K_{d}=\frac{\Delta S}{R}-\frac{\Delta H}{RT}$$

where: *K_d_* is the Langmuir equilibrium constant (L/g); *T* is the temperature (K); *R* is the gas constant (8.314 kJ/mol K). The Δ*S* and Δ*H* values can be, respectively, attained from the intercept and slope of plot ln *K_d_* versus 1/*T* (Figure 15). The Van’t Hoff equation relates the coefficient of distribution with Δ*S* and Δ*H* at a given temperature. The negative values of Δ*G* at 30 °C, 40 °C, and 50 °C (Table 4), infer that Cu(II) adsorption onto various beads was thermodynamically possible and spontaneous for all of the studied temperatures. The positive enthalpy demonstrates the adsorption endothermic nature.

### 3.9. Adsorption Mechanism

The FTIR spectra of the adsorbents (Figure 4) show peaks due to −OH, C=O, and COO^−^ stretching and Fe−O vibrations which come from the active binding sites for Cu(II) adsorption. Additionally, the maximal adsorption for the AVMNCABs, AVCABs, and CABs values are observed at pH 4. The point of zero charge (pH_pzc_) is 3.72 for AVFMNPECABs and AVCABs and 3.32 for CABs (Figure 9). The surface is negatively charged above pH_pzc_, which facilitates the electrostatic attraction with the Cu(II) ion. 

### 3.10. Comparison with Other Adsorption Methods

The adsorption efficiencies of the synthesized bio-sorbents were compared with the values obtained from the literature for Cu(II) removal using other materials (Table 5). It was found that our materials showed better performance, as evidenced by the adsorption capacity values. Furthermore, our functionalized adsorbents showed a greater affinity for Cu(II) compared to the non-functionalized adsorbents.

## 4. Conclusions

The synthesized bio-sorbents CABs, AVCABs, and AVMNCABs, can be used as cost-efficient and environmentally friendly materials for removing Cu(II) from water. The adsorption studies demonstrate the applicability of the developed spectrophotometric method. The adsorption results of CABs, AVCABs, and AVMNCABs follow a Langmuir model with a maximal adsorption capacity of 15.38 mg/g, 41.66 mg/g, and 111.11 mg/g, respectively, with the sequence: AVMNCABs > AVCABs > CABs. The kinetic data indicate that the adsorption of Cu(II) on the synthesized adsorbents follows a pseudo-second-order model. Thermodynamic studies reveal the spontaneous and endothermic nature of the adsorption process. The beads demonstrate high recyclability, and the desorption efficiencies of CABs, AVCABs, and AVMNCABs were 92.7%, 96.4%, and 98.6%, respectively, on the 7th cycle. 

## Figures and Tables

**Table 1:** Average crystal size of CABs, AVCABs, and AVMNCABs calculated using Debye–Scherrer’s formula.

Samples	Most Intense Peak (2*θ*, Degrees)	Most Intense Peak (*θ*, Degrees)	FWHM of the Most Intense Peak (*β*, Radians)	Particle Size (D, nm)
CABs	36.61	18.30	2.00	0.072
AVCABs	36.25	18.12	1.11	0.131
AVMNCABs	33.11	16.55	6.57	0.022

**Table 2:** Parameters of the adsorption isotherm of Cu(II) on various adsorbents.

Isotherm	Value of Parameters			
**Langmuir**	*q_max_* (mg g^−1^)	*K_L_*	R^2^	*R_L_*
CABs	15.38	0.193	0.982	0.518
AVCABs	41.66	0.049	0.991	0.671
AVMNCABs	111.11	0.014	0.997	0.877
**Freundlich**	*K_F_* (mg g^−1^) (mg L^−1^)*^n^*	*n*	R^2^	
CABs	3.296	1.182	0.959	
AVCABs	2.137	1.05	0.986	
AVMNCABs	1.62	1.026	0.994	
**Temkin**	*B*_1_	*K_T_* (L mg^−1^)	R^2^	
CABs	28.19	0.058	0.779	
AVCABs	30.99	0.03	0.837	
AVMNCABs	17.26	0.091	0.919	

**Table 3:** Kinetic parameters for Cu(II) adsorption on various adsorbents.

Models	Kinetics Parameters		
**Pseudo-First-Order**	*k*_1_ (min^−1^)	*q_e_* (mg g^−1^)	R^2^
CABs	0.027	426.578	426.578
AVCABs	0.041	426.578	426.578
AVMNCABs	0.043	426.578	426.578
**Pseudo-Second-Order**	*k*_2_ (g mg^−1^ min^−1^)	*q_e_* (mg g^−1^)	R^2^
CABs	0.092	0.471	0.931
AVCABs	0.129	0.512	0.973
AVMNCABs	0.312	0.510	0.997
**Intra-particle Diffusion**	*k_d_* (mg g^−1^ min^−1^)	*C* (mg g^−1^)	R^2^
CABs	0.035	0.040	0.980
AVCABs	0.036	0.097	0.991
AVMNCABs	0.027	0.226	0.949
**Elovich model**	*α* (mg g^−1^ min^−2^)	*β* (g mg^−1^ min^−1^)	R^2^
CABs	−0.042	0.090	0.900
AVCABs	0.005	0.095	0.962
AVMNCABs	0.160	0.069	0.991

**Table 4:** Thermodynamic parameters for Cu(II) adsorption on CABs, AVCABs, and AVMNCABs.

Adsorbents	Δ*H* (kJ/mol)	Δ*S* (J/mol/K)	Δ*G* (kJ/mol)		
			303 K	313 K	323 K
CABs	0.82	215.7	−8.6	−12.7	−14.6
AVCABs	0.8	210.2	−8.21	−12.17	−14.07
AVMNCABs	0.57	153.2	−6.6	−9.4	−10.9

**Table 5:** Adsorption capacities of different adsorbents—comparison with literature.

S.No.	Adsorbents	Adsorption Capacity (mg g^−1^)	pH	Ref.
1.	Polyaniline/calcium alginate composite	79.0	3.0	[67]
2.	Magnetic composite gel beads (CMC/SA/graphene oxide@Fe_3_O_4_)	55.96	5.0	[68]
3.	Nanochitosan/sodium alginate/microcrystalline cellulose beads	43.3	5.0	[69]
4.	Fluidized zeolite beads	23.3		[70]
5.	Chitosan nanoparticles-bentonite-alginate	12.21	7.0	[71]
6.	γ-Fe_2_O_3_ nanoparticles	34.0		[72]
7.	Iminodiacetic acid-functionalized Paeonia ostii seed coats	36.6	5.0	[73]
8.	Plasma-modified activated carbon	21.4	5.0	[74]
9.	Oxidized Functionalized multiwalled carbon nanotubes	14.086		[75]
10.	Sewage sludge-based composite adsorbent diethylenetriaminepentaacetic acid	31.42	3.0	[76]
11.	(a) Calcium alginate beads (CABs)	15.38	4.0	Present study
	(b) Aloe-vera calcium alginate beads (AVCABs)	41.66	4.0	Present study
	(c) Aloe-vera functionalized magnetic nanoparticles entrapped calcium alginate beads (AVMNCABs)	111.11	4.0	Present study

## Data Availability

Data will be made available upon request.
